# Negotiating Medical Insurance Drug Prices: The Role in Reducing Costs of Orphan Drugs for Rare Diseases

**DOI:** 10.34172/ijhpm.2023.8195

**Published:** 2023-11-13

**Authors:** Jinmiao Lu, Xiaohua Ying, Zhiping Li

**Affiliations:** ^1^Department of Clinical Pharmacy, National Children’s Medical Center, Children’s Hospital of Fudan University, Shanghai, China; ^2^NHC Key Laboratory of Health Technology Assessment, Department of Health Economics, School of Public Health, Fudan University, Shanghai, China

## Dear Editor,

 There are 20 million patients with rare diseases in China, and over 200 000 new patients are diagnosed each year. In 2018, the Chinese government identified the first batch of rare diseases, which included 121 conditions. Drugs developed for rare diseases (orphan drugs) are often prohibitively expensive for most families, making their availability and affordability a recurring topic in literature.^1^ The global challenge of ensuring equitable access to treatment for rare diseases has gained much attention.

 Reducing out-of-pocket drug costs can improve compliance and affordability.^2^ Countries have implemented policies to reduce orphan drugs, such as creating triggers to end market exclusivity and imposing windfall profit taxes. In Europe, government intervention in pharmaceutical markets, such as price control policies, has been effective.^3^ The Orphan Drug Act of 1983 in the US pharmaceutical companies to invest more in research and development for orphan drugs.^4^

 China’s healthcare system negotiates drug prices to address this issue, resulting in steep price cuts, averaging over 60%. The extent of the price cuts may have implications for drug companies’ global pricing systems, prompting China’s health insurance administration to introduce price secrecy regulations.

 Specifically, according to the Chinese government’s regulations, the cost-sharing ratios for rare disease drugs that have successfully undergone negotiations are as follows:

 First, for rare disease drugs with costs up to 300 000 yuan, the government covers 80% of the expenses, and the individual is responsible for paying the remaining amount.

 Second, for rare disease drugs with costs between 300 000 and 700 000 yuan (including 700 000 yuan), the government covers 85% of the expenses, and the individual is responsible for the remaining amount. Third, for rare disease drugs with costs exceeding 700 000 yuan, the government covers 90% of the expenses, and the individual is responsible for the remaining amount. The specific cost-sharing ratios are based on different ranges of drug costs to ensure that patients receive appropriate financial support. The Chinese government’s reimbursement ratio and the substantial cost of rare disease drugs necessitate pharmaceutical manufacturers to lower prices as a prerequisite for inclusion in the medical insurance reimbursement system.

 We present the annual cost reductions for all 27 rare-disease drugs before and after Medicare negotiations (Table). Over half of the companies that received price reductions in China chose to keep their health insurance prices confidential.

 To assess the decline in rare-disease drugs, we used a World Health Organization (WHO)-defined single-family annual disposable income.^5^ The calculation method of multiple catastrophic spending (MCS) involves taking the yearly total medication expenses for a specific rare disease drug on a standard adult weighing 70 kg and dividing it by the average catastrophic spending of Chinese individuals. The resulting value is the multiple obtained. At last, the price declined by 65% on average, with seven drugs transitioning from catastrophic to affordable. For example, SPINRAZA^TM^ experienced a price drop of up to 95% following health insurance negotiations. Similarly, American health insurance plans began negotiating drug prices in 2026.^6^

 However, this policy can also have adverse effects, such as manufacturers withdrawing their products from the insurance market and limiting patients’ access to medicine. In China, health insurance negotiations mainly aim to include drugs in patent or exclusive use, which may limit patients’ drug choices. In this study, the 27 orphan drugs belonged to only 15 rare diseases, focusing on only a few specific conditions (Table). Moreover, patients with rare diseases face practical challenges in accessing drugs, such as limited availability in local hospitals and inconsistent health insurance requirements nationwide. According to the Chinese drug-to-hospital policy, the expense incurred on medication surpass at most 30% of the overall expenses. In the long term, China’s healthcare reform may have a limited impact on drug spending.^7^

**Table:** Information of 27 Orphan Medical Products Corresponding to 15 Rare Diseases

**Rare Diseases**	**Morbidity (‱)**	**Generic Name**	**MCS** **Before**	**MCS** **After**	**Difference (%)**
Hyperphenylalaninemia	0.80	Sapropterin	16.96	15.54	-8.37
Homozygous familial hypercholesterolemia	0.03	Ezetimibe	0.38	0.17	-55.26
		Evolocumab	1.45	0.31	-78.62
Multiple sclerosis, MS	0.50	Dimethyl fumarate	9.69	6.78	-30.03
		Ofatumumab	8.54	5.51	-35.48
		Teriflunomide	4.77	0.88	-81.55
Idiopathic pulmonary arterial hypertension	0.50	Treprostinil	8.11	3.48	-57.09
		Ambrisentan	1.96	0.35	-82.14
		Bosentan	1.57	0.33	-78.98
		Riociguat	3.23	0.46	-85.76
Gaucher disease	0.10	Imiglucerase	150.89	121.44	-19.52
Amyotrophic lateral sclerosis	0.30	Riluzole	0.47	0.13	-72.34
Spinal muscular atrophy	1.00	Risdiplam	129.67	7.45	-94.25
		Nusinersen	142.27	6.10	-95.71
Tuberous sclerosis complex	0.50	Everolimus	3.26	0.81	-75.15
Niemann-Pick disease	0.10	Miglustat	9.72	2.39	-75.41
Parkinson’s disease (young-onset, early-onset)	3.00	Levodopa/Carbidopa	0.52	0.14	-73.08
		Ropinirole	0.50	0.15	-70.00
		Droxidopa	0.58	0.16	-72.41
		Rasagiline	0.48	0.1	-79.17
Neuromyelitis optic spectrum disorder	0.20	Inebilizumab	9.04	2.71	-70.02
Glycogen storage disease (type I, II)	0.20	Alglucosidase alfa	103.15	88.71	-14.00
Idiopathic pulmonary fibrosis	1.50	Pirfenidone	2.79	0.75	-73.12
		Nintedanib	8.15	2.24	-72.52
Hemophilia	0.30	Recombinant human coagulation factor VIIa	36.43	8.23	-77.41
		Recombinant human coagulation factor IX	64.18	17.64	-72.51
Hereditary angioedema	0.50	Lanadelumab	153.39	38.74	-74.74

Abbreviation: MCS, Multiple catastrophic spending. Source: https://www.smpaa.cn/.

 In conclusion, medical insurance negotiations help reduce drug prices, lessen patient burdens, and promote the rational use of medicine. Negotiating drug prices for rare diseases is a favorable policy that can lower drug costs and improve medication access. It is essential to consider the impact of such negotiations on n developing innovative drugs and the variety and quality of available drugs.

## Ethical issues

 Not applicable.

## Competing interests

 Authors declare that they have no competing interests.
